# Genome-wide analysis of SSR and ILP markers in trees: diversity profiling, alternate distribution, and applications in duplication

**DOI:** 10.1038/s41598-017-17203-6

**Published:** 2017-12-20

**Authors:** Xinyao Xia, Lin Lin Luan, Guanghua Qin, Li Fang Yu, Zhi Wei Wang, Wan Chen Dong, Yumin Song, Yuling Qiao, Xian Sheng Zhang, Ya Lin Sang, Long Yang

**Affiliations:** 1 College of Forestry, College of Plant Protection, College of Life Sciences, State Key Laboratory of Crop Biology, Agricultural Big-Data Research Center, Shandong Agricultural University, Tai’an, 271018 China; 2 Shandong Academy of Forestry, Jinan, 250014 China

## Abstract

Molecular markers are efficient tools for breeding and genetic studies. However, despite their ecological and economic importance, their development and application have long been hampered. In this study, we identified 524,170 simple sequence repeat (SSR), 267,636 intron length polymorphism (ILP), and 11,872 potential intron polymorphism (PIP) markers from 16 tree species based on recently available genome sequences. Larger motifs, including hexamers and heptamers, accounted for most of the seven different types of SSR loci. Within these loci, A/T bases comprised a significantly larger proportion of sequence than G/C. SSR and ILP markers exhibited an alternative distribution pattern. Most SSRs were monomorphic markers, and the proportions of polymorphic markers were positively correlated with genome size. By verifying with all 16 tree species, 54 SSR, 418 ILP, and four PIP universal markers were obtained, and their efficiency was examined by PCR. A combination of five SSR and six ILP markers were used for the phylogenetic analysis of 30 willow samples, revealing a positive correlation between genetic diversity and geographic distance. We also found that SSRs can be used as tools for duplication analysis. Our findings provide important foundations for the development of breeding and genetic studies in tree species.

## Introduction

Perennial trees constitute more than 50% of the terrestrial biodiversity, act as large and persistent carbon sinks, and play important roles in climate regulation^1^. They also give rise to wood resources which provide raw materials for human essential needs^2^. Besides, many tree species offer special industrial material. For example, *Hevea brasiliensis* produces natural latex rubber which is a valuable material for medicine and industry^3^, and *Theobroma cacao* supplies raw materials for the production of chocolate^4^. Yet despite their great value, progress in breeding and molecular study has been hampered by their inherent long growth cycles, high levels of heterozygosis, and complex reproduction.

The use of molecular markers is increasingly important in breeding^5^. Simple sequence repeats (SSRs), also known as microsatellites or short tandem repeats, are segments of DNA with a basic repeat unit of fewer than seven base pairs^6^. SSRs are widely distributed in eukaryotic genomes and have been extensively applied in genetic studies and breeding programs^7^. In recent years, genetic studies of tree species have been advanced by the development and application of SSR markers.

Introns are non-coding sequences distributed in eukaryotic genomes between exons, and are exposed to low selective pressure^8^. Previous studies suggested that intron sequences evolve much faster and contain more polymorphisms than exons^9^. These characteristics introduce them as desirable polymorphic molecular markers. In recent years, intron length polymorphism (ILP) markers have been successfully used for the construction of genetic maps^10^, species identification^11^, and large-scale genotyping analyses^8^. Identifying suitable introns is the key point to ILP marker development, and this is facilitated by the availability of complete genome data for model organisms. By comparing expressed sequence tags (ESTs) or coding DNAs with the genome sequence of model plants, the intron positions of species without available genome sequences can be predicted and used for developing potential intron polymorphism (PIP)^12^. ILPs and PIPs are usually defined together as intron polymorphism (IP).

With the progression of next-generation sequencing, an increasing number of tree genome sequences have become available^13–15^, which provide the foundations for the development and application of molecular markers. In this study, we performed a genome-wide identification of SSR, ILP, and PIP markers in 16 tree species whose genome sequences are currently available. We used these markers to perform phylogenetic analysis in 30 willow samples, and duplication analysis in *Populus trichocarpa* and *Elaeis guineensis*. The results will be useful in modern molecular biology and genetic diversity studies.

## Results

### SSR and ILP loci

Using the Perl pipeline, 67,259,820 SSR loci were identified from 16 tree species (Table 1), and genome size was found to be positively correlated with the number of identified SSR loci. Two pine species, *Pinus taeda L*. and *Pinus lambertiana* whose genome sizes accounted for 62.42% of the analysed species, contained 67.99% (45,732,066) of the total SSR loci, while *Prunus persica* possessed the smallest genome and contained the fewest SSR loci. By contrast, a negative correlation between genome size and the density of SSR loci was revealed. The lowest SSR density (385 per Mb) was found in *Picea abies*, which possesses a large genome (11.7 Gb). *Morus notabilis* possesses a relatively small genome (0.3 Gb), but exhibited the largest SSR loci density (2,272 per Mb). These results suggest that the application of SSR markers may be more efficient in small genomes because of the higher loci density.

**Table 1:** Seven motifs of SSR loci in 16 species.

Species	Monomer	Dimer	Trimer	Tetramer	Pentamer	Hexamer	Heptamer	All	Size (Mb)	Density
*P. persica*	27,034	40,294	23,975	31,782	12,441	178,105	58,671	372,302	219	1,700
*S. babylonica*	31,055	57,451	51,276	50,049	20,208	254,705	86,345	551,089	295	1,868
*J. curcas*	83,074	33,951	32,392	42,609	12,737	227,431	78,032	510,226	308	1,657
*M. notabilis*	111,688	72,963	42,208	48,430	24,611	279,875	128,953	708,728	312	2,272
*T. cacao*	32,673	35,792	30,515	38,622	14,538	250,827	80,076	483,043	334	1,446
*P. trichocarpa*	59,920	54,354	57,563	60,302	26,344	336,295	124,341	719,119	403	1,784
*P. euphratica*	63,381	65,147	118,408	82,122	29,200	432,937	153,442	944,637	480	1,968
*P. dactylifera*	35,953	61,436	49,823	55,589	21,096	324,903	103,674	652,474	547	1,193
*A. trichopo*	100,331	159,465	65,405	69,895	26,679	469,772	185,400	1,076,947	682	1,579
*F. excelsior*	109979	52,036	54,512	84,842	37,870	499,268	169,920	1,008,427	846	1,192
*H. brasiliensis*	78,165	103,931	107,094	154,575	69,316	827,752	280,723	1,621,556	1,362	1,191
*E. guineensis*	78, 323	105,403	85,834	108,984	43,820	747,818	265,081	1,435,263	1,485	967
*G. biloba*	356,257	893,397	319,060	747,079	99,851	3,423,774	1,065,480	6,904,898	10,220	676
*P. abies*	333,417	227,946	306,955	341,052	100,714	2,501,615	805,669	4,617,368	11,980	385
*P. taeda*	570,852	840,323	1,399,969	1,507,006	401,640	10,793,943	3,524,504	19,038,237	21,709	877
*P. lambertiana*	862,648	1,435,961	1,701,344	1,895,896	625,256	15,553,791	4,618,933	26,693,829	27,238	980
Total	2,856,427	4,239,850	4,446,333	5,318,834	1,566,321	37,102,811	11,729,244	67,259,820	78,420	
Percentage	4.25%	6.30%	6.61%	7.91%	2.33%	55.16%	17.44%	100.00%		

SSR loci can be divided into seven types, from monomers to heptamers according to motif length. In this study, hexamers were the most abundant type, accounting for 55.16% of all motifs, followed by heptamers (17.44%). Pentamers were the least abundant type (2.33%). For separate loci type, the proportions fluctuated within a narrow range among most species (Supplementary Table S1).

We next extracted the two SSR loci types with the highest frequency from each species (Table 2). AT/TA base pairs were found to be the most prevalent dimers, followed by AG/TC. AAT/TTA were the most frequent trimer motif, followed by AAG/TTC, while the most frequent tetramer, pentamer, hexamer, and heptamer motifs were AAAT/TTTA, AAAAT/TTTTA, AAAAAT/TTTTTA, and AAAAAAT/TTTTTTA, respectively. A/T bases were shown to make up the majority of base pairs in SSR loci with the highest frequencies. We further analysed the base pair composition in all identified SSR loci (Supplementary Table S2), revealing that the number of A/T base pairs was more than twice that of G/C base pairs in 10 species. In other six species, A/T to G/C ratios were ≥3, while in *Populus euphratica Oliv*, this ratio was up to 4.65. These results indicate that A/T comprised a significantly larger proportion than G/C of the base pair composition in identified SSR loci.

**Table 2:** The top two SSR loci in 16 species.

Species	Dimer		Trimer		Tetramer		Pentamer		Hexamer		Heptamer	
*P. persica*	at	ga	aat	tct	ttta	ataa	tttta	ataaa	ttttta	aaaaag	tttttta	aaaaaag
	8,128	5,712	1,911	1,340	3,057	1,396	753	410	2,103	1,992	885	590
*S. babylonica*	ta	ag	aat	ata	aaat	aata	aaaat	tattt	aaaaat	aaaata	taaaaaa	tattttt
	17,709	3,981	7,020	3,712	5,622	2,509	1,852	705	6,771	4,906	3,004	2,476
*J. curcas*	at	ag	aat	tat	aaat	ttat	aaaat	aaaag	aaaaat	aatttt	aaaaaat	aaaataa
	11,973	2,334	4,045	2,114	4,458	2,590	961	552	3,717	2,267	1,069	898
*M. notabilis*	at	ag	aat	ata	aaat	ttat	aaaat	aaaag	aaaaat	aaattt	tttttta	tttttat
	24,281	6,800	5,359	2,130	5,327	2,293	3,227	1,035	6,366	6,074	2,154	1,735
*T. cacao*	at	ag	aat	gaa	ttta	ataa	taaat	aaaat	aatatt	aaaata	tttttta	aataaaa
	13,652	2,662	3,415	1,973	3,203	1,764	1,243	1,055	6,409	4,497	819	704
*P. trichocarpa*	at	ag	aat	aag	aaat	aata	aaaat	aaaag	ttttta	aaaata	taaaaaa	tattttt
	17,008	3,897	6,303	3,200	6,203	2,625	2,278	961	9,395	4,431	5,772	4,179
*P. euphratica*	at	ag	aat	tat	ttta	tttc	aaaat	aaaag	ttttta	tgatct	taaaaaa	tattttt
	19,436	5,284	12,436	8,007	8,988	4,459	3,201	1,266	11,915	6,163	5,772	4,179
*P. dactylifera*	at	ag	ttc	aat	aaat	tttc	aaaat	aaaag	atattg	atcact	aaaaaat	aaaaaag
	11,336	8,729	2,897	2,794	2,853	2,108	1,165	885	168	126	931	844
*A. trichopo*	ag	ta	aat	tct	aaat	aata	aaaat	ttatt	aaaaat	tactag	aaaaaat	agagaga
	31,649	17,769	4,848	3,753	9,134	2,980	2,553	1,102	9,056	4,873	3,754	3,620
*F. excelsior*	at	tc	aat	ttc	aaat	aatt	aaaat	aaaag	aaaaat	tttatt	aaaaaat	aaaaata
	10,959	5,554	6,699	2,339	9,138	6,673	2,838	818	10,401	3,899	3,613	1,884
*H. brasiliensis*	at	ag	aat	ata	aatt	aaat	ttttc	aaaat	aaaaat	ttaatt	aaatttt	tttttta
	28,830	10,481	12,541	6,997	14,319	11,057	4,019	3,643	11,402	10,792	3,558	2,546
*E. guineensis*	at	ag	aag	tct	aaat	ataa	ttttc	aaaat	aaaaat	aaaaag	aaaaaat	tattttt
	25,047	13,311	6,914	6,818	6,235	4,283	1,994	1,808	12,206	6,355	3,997	2,255
*G. biloba*	at	tg	aag	aat	tatg	aaat	aaaat	ttatt	tatatg	aaaaat	aaaaaat	actttaa
	267,740	114,986	27,247	23,099	80,939	34,704	7,732	4,378	44,881	39,674	10,412	8,622
*P. abies*	at	tg	aat	aag	aaat	ttaa	aaaat	tttat	aaaaat	aaaata	aaaaaat	aaaaata
	64713	20653	20346	15799	17897	15200	3887	2072	22374	13662	5766	3630
*P. taeda*	at	ga	aat	ttc	aaac	ttta	aaaat	tgtat	aaaaat	ataatg	taaaaca	tgtttta
	184,257	93,952	94,526	89,987	110,137	86,787	17,964	14,389	112,758	92,895	111,036	60,290
*P. lambertiana*	at	tc	aat	ttc	aaat	aata	aaaat	aaata	aaaaat	taacct	aaaaaat	aaaaata
	472,134	99,937	139,649	102,041	147,638	83,716	41,319	15,814	191,720	147,599	50,732	35,760

To identify sufficient ILP markers, the screening conditions were set mildly with no length limits. Because six of the species analysed lacked gene position information, which was not appropriate for ILP identification, a total of 3,811,360 ILP loci were obtained from the remaining 10 species (Supplementary Table S3). Compared with SSR loci, the number of ILP loci was much smaller for each analysed species, ranging from 193,575 (*M. notabilis*) to 656,824 (*Populus euphratica*) with fewer differences in number among species. Similar to SSR loci, the genome size exhibited a negative correlation with the ILP loci density. However, the variation in ILP loci density among different species, ranging from 352 per Mb (*Amborella trichopo*) to 1,513 per Mb (*T. cacao*), was larger than that in SSR loci.

### SSR, ILP, and PIP markers

A total of 530,614 SSR, 267,636 ILP, and 11,872 PIP markers were identified from 16 analysed species (Supplementary Table S4). Detailed information of these markers can be downloaded from our database (http://biodb.sdau.edu.cn/xxyssr/result_data.zip). The number of SSR markers ranged from 21,442 (*Jatropha curcas*) to 70,442 (*E. guineensis*), and was positively correlated with the genome size of analysed species. However, the number of ILP markers did not show an obvious correlation with genome size. This may be explained that ILP markers were located in gene coding regions whereas SSRs were distributed genome-wide. Therefore, the number of ILP markers should be related to the number of genes in the genome. By comparing available EST sequences against model plant genomes (*Arabidopsis* and *Oryza sativa*), we predicted the existence of 11,872 PIP markers from *H. brasiliensis* and *Pinus taeda*. This number is far less than that of SSR and ILP markers, which may reflect divergence among analysed and model species.

### Distribution patterns of ILP and SSR markers

**Figure 1:**
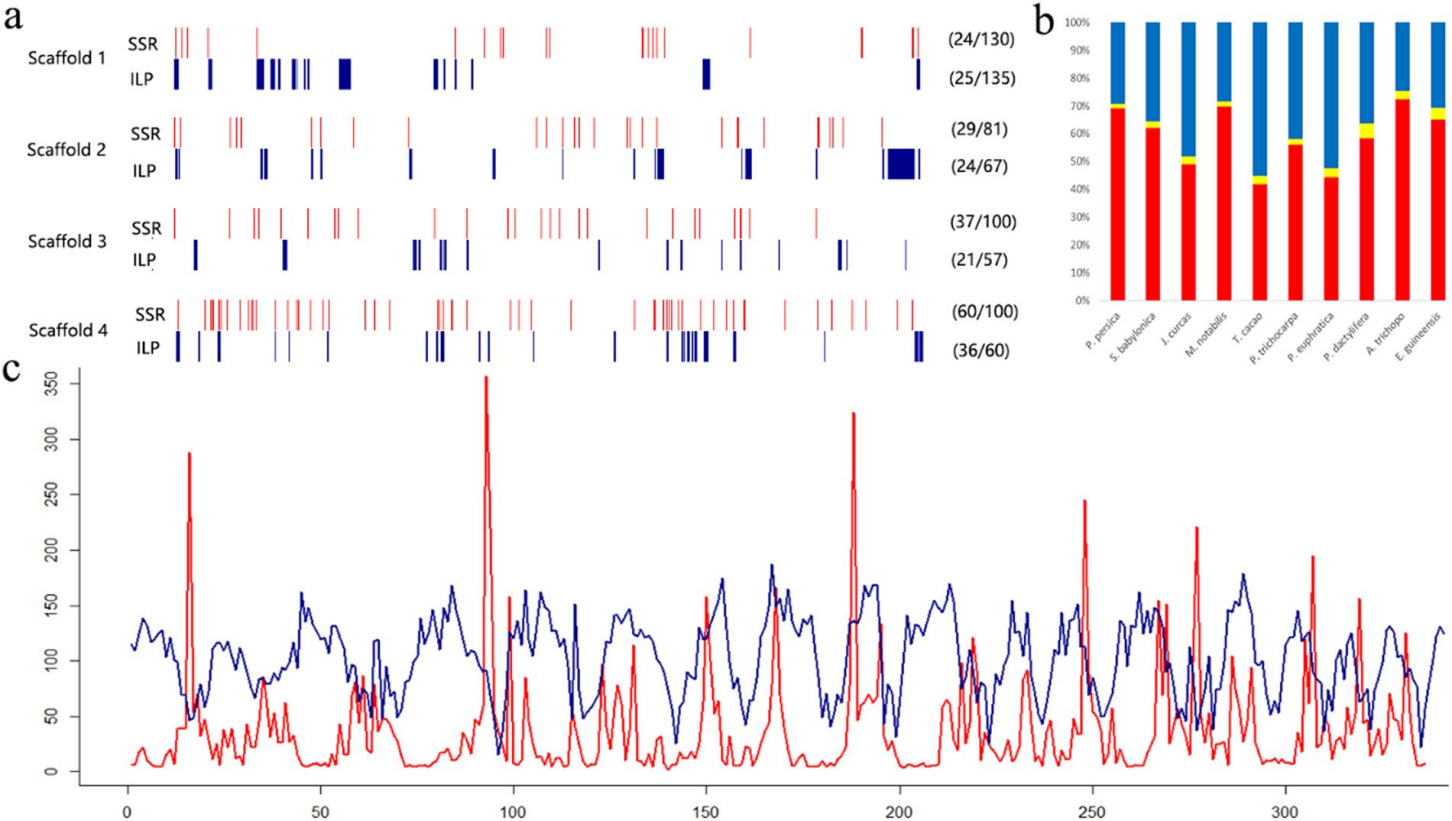
Distribution feature of the molecular markers. (**a**) Distribution of SSR and ILP markers. Four scaffolds were randomly selected from the genome of *Morus notabilis*. Red and blue lines indicate SSR and ILP markers, respectively. Numbers on the right side represent the number and density of markers. (**b**) Proportion of the concomitant and separated distribution of SSR and ILP markers. Red and blue columns represent the separated SSR and ILP markers, respectively. Yellow columns represent the concomitant markers. (**c**) Density of SSR loci and gene coding sequences across the genome of *Populus trichocarpa*. Red line represents the density of SSR loci and blue line represents that of gene coding sequences.

We constructed a distribution map of SSR and ILP markers by randomly selecting four *M. notabilis* scaffolds (Fig. 1a). The number of SSR markers on the selected scaffolds ranged from 24 (Fig. 1a, scaffold 1) to 60 (Fig. 1a, scaffold 4), and the density ranged from 81 per Mb (Fig. 1a, scaffold 2) to 130 per Mb (Fig. 1a, scaffold 1). The number of ILP markers ranged from 21 (Fig. 1a, scaffold 3) to 36 (Fig. 1a, scaffold 4), and the density ranged from 57 per Mb (Fig. 1a, scaffold 3) to 135 per Mb (Fig. 1a, scaffold 1). The distribution map showed that the SSR markers were sparsely and unevenly distributed on the scaffolds. They often appeared as lines on the map because of their limited length. Conversely, the large span of introns made ILP markers appear as bar plots. The map showed a concomitant and alternate distribution pattern of SSR and ILP markers in certain sections of all analysed species (Fig. 1b). However, the concomitant distribution rates were relatively low, ranging from 2.35% to 8.10%. In *Prunus persica*, only 637 (2.35%) SSR markers intersected with ILP markers (Supplementary Table S5). These results indicated a mutual independence between SSR and ILP markers.

### SSR polymorphisms

**Figure 2:**
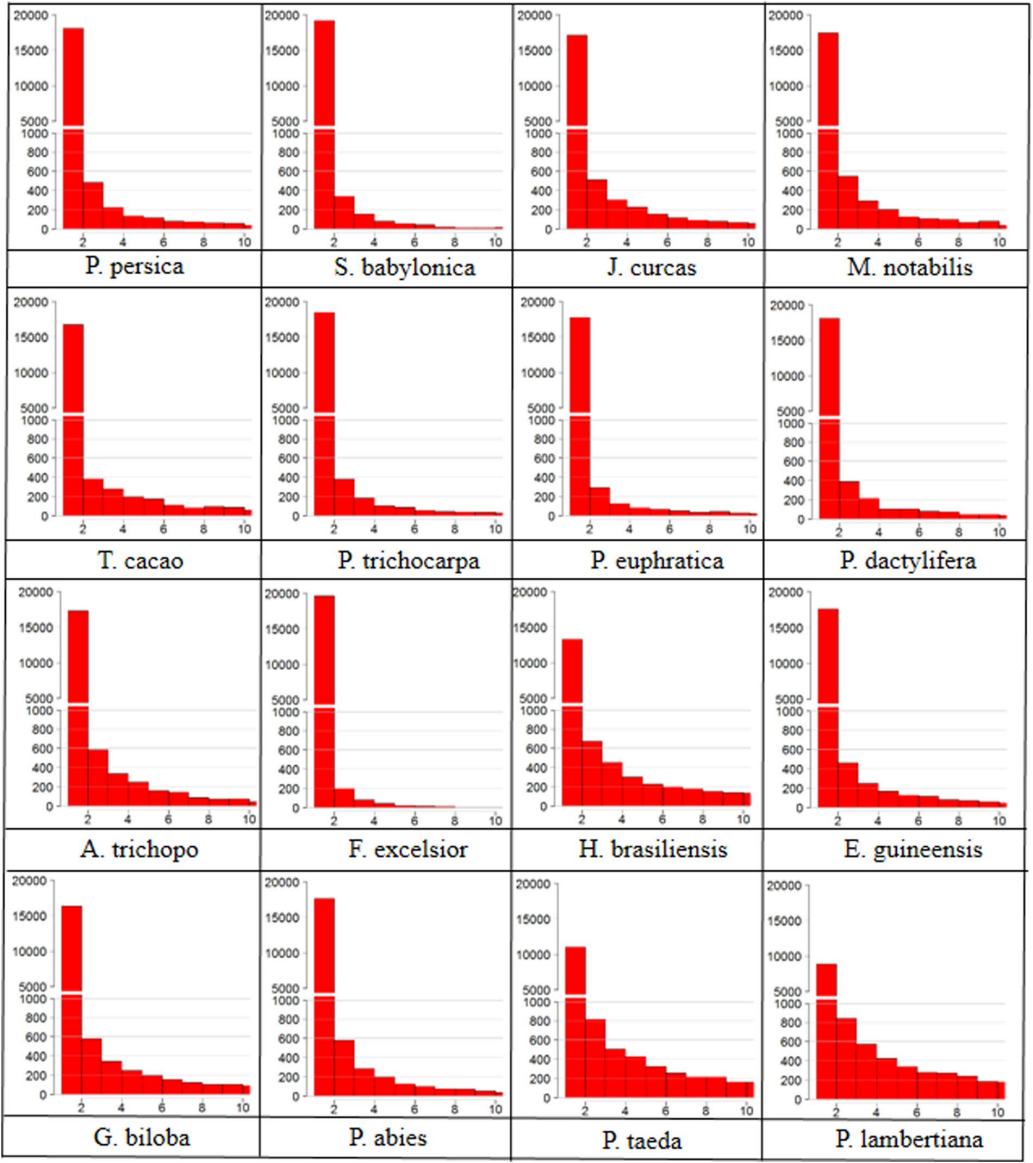
Schematic of the content of monomorphic and polymorphic markers. The first red bar shows the number of monomorphic markers and other bars represent the polymorphic markers which amplified two, three, or more bands by e-PCR.

To examine SSR polymorphisms, 20,000 markers of each species were randomly selected and electronically amplified in their own genomes. After the calculation of amplification sites, the number of monomorphic and polymorphic markers was depicted using a histogram (Fig. 2). Among all the amplification sites, monomorphic markers comprised the largest proportion (average proportion 75.56%, Supplementary Table S6). The proportions of polymorphic markers were limited and were positively correlated with genome size. In the 10 species with genomes smaller than 1 Gb, the proportions of polymorphic markers were < 20% (Supplementary Table S7). However, in *Ginkgo biloba* and *Picea abies* which possess genomes >10 Gb, polymorphic markers comprised 24.7% and 22.6%, respectively. In *Pinus taeda* and *Pinus lambertiana*, whose genomes were >20 Gb, the polymorphic markers accounted for 63.5% and 52.7%, respectively. We also found that the proportions of polymorphic markers were positively associated with the contents of repetitive sequences. In the six species whose genomes contain about 45% repetitive sequences (*Prunus persica*, *J. curcas*, *M. notabilis*, *T. cacao, Populus trichocarpa*, and *Populus euphratica*), polymorphic markers accounted for proportions of around 20%. By contrast, the contents of polymorphic markers were higher than 50% in *Pinus taeda* and *Pinus lambertiana*, where repetitive sequences took up more than 80% of the genome.

### Phylogenetic analysis of willow samples

**Figure 3:**
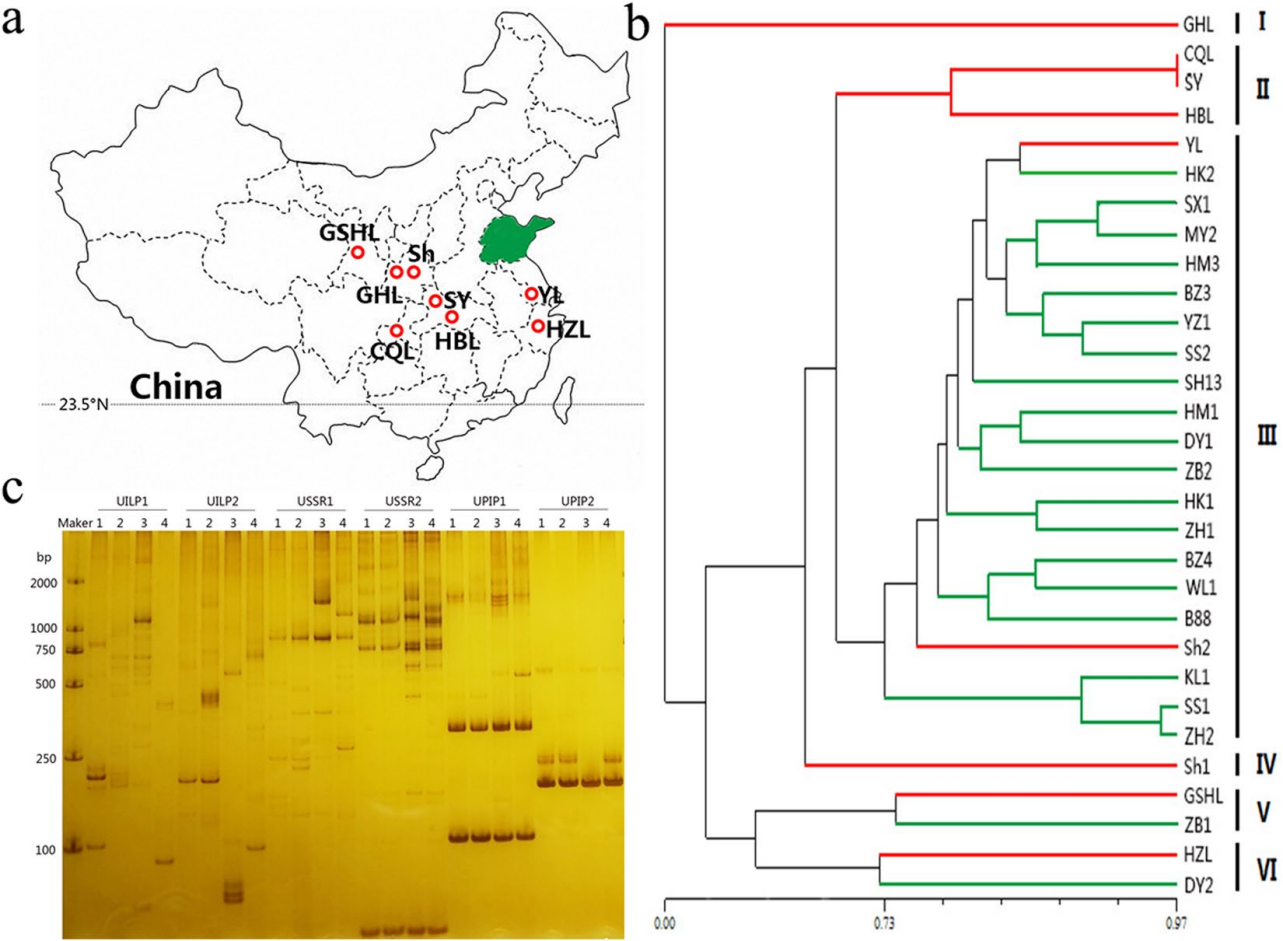
Verification of markers. (**a**) Locations of willow samples used in phylogenetic analysis. Contour map of sampling places. Red circles denote the position of sampling sites. Shandong Province is highlighted in green. The skeleton map was constructed by R package “maps”, then modified using Adobe Photoshop (version 14.0, X64). (**b**) UPGMA-based phylogenetic tree of the 30 willow samples. Numbers on each node are bootstrap values of 1,000 replicates. Green branches indicate the samples located in Shandong Province. (**c**) Verification of the universal markers. PCR products of the markers were separated by electrophoresis using 6% non-denaturing polyacrylamide. Lanes 1–4 represent the four plant species *S. babylonica*, *Populus trichocarpa*, *M. notabilis*, and *Selaginella*, respectively. The gel presented in panels (**c**) was cropped, and the exposure was adjusted.

To evaluate the efficiency of the molecular markers identified in this study, we performed the phylogenetic analysis of 30 willow samples. The sampling locations were marked on the map (Fig. 3a). Five SSR markers and six ILP markers were randomly selected and used for PCR amplification to construct an Unweighted Pair Group Method with Arithmetic mean (UPGMA)-based phylogenetic tree (Fig. 3b). This clustered the 30 willow samples into six groups. Samples CQL, SY, and HBL, which all derived from southwest China, were clustered in Group II, while 19 of 21 samples from Shandong province were clustered in Group III. Group I and Group IV each contained only one sample, which was distinct from the other samples. Two samples far apart from each other were clustered together in Group V, and similar conditions were found in Group VI.

### Development of universal markers

To develop universal markers, all the obtained markers were examined in 16 analysed species by electronic amplification. A marker was assessed as universal if its primers successfully amplified loci in all 16 species. A total of 54 SSR, 418 ILP, and four PIP markers were identified as universal markers. To evaluate the efficiency of these markers, two ILP, two SSR, and two PIP markers were randomly selected and PCR-amplified in four species (*Salix babylonica*, *Populus trichocarpa*, *M. notabilis*, and *Selaginella*) (Fig. 3c). As a result, each selected marker amplified a series of fragments of different lengths in every analysed species. Compared with PIP markers, ILP and SSR markers amplified more fragments, so presented with higher levels of polymorphism. The bands obtained from different marker types exhibited an alternative distribution pattern, suggesting the potential efficiency of the combined use of these universal markers.

### Duplication analysis of *Populus trichocarpa* and *E. guineensis*

We next determined whether SSR markers could be used for duplication analysis by analysing the distribution of SSRs and genes in the *Populus trichocarpa* genome (Fig. 1c). Both SSRs and gene sequences were evenly distributed, and exhibited an alternative pattern throughout the genome. In general, only 5.6% (7586) of SSRs were located in gene coding regions. The alternative distribution pattern suggested that SSR markers could be used for duplication analysis with the intergenic regions.

**Figure 4:**
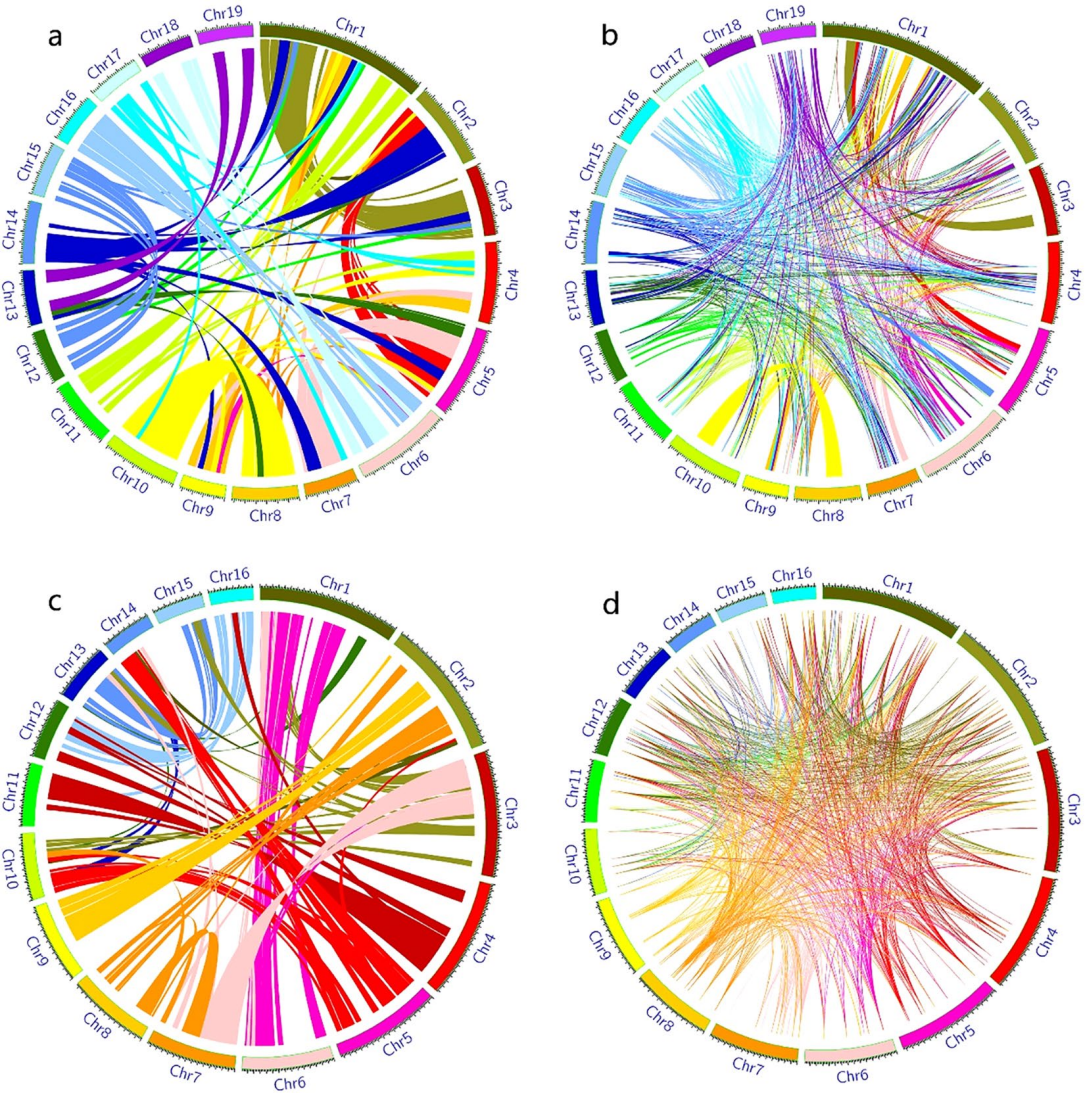
Duplication analysis in *Populus trichocarpa* and *E. guineensis*. Duplication analyses were performed based on gene coding sequences (**a**) and SSR markers (**b**) in *Populus Trichocarpa*, and on gene coding sequences (**c**) and SSR markers (**d**) in *E. guineensis*.

We then performed duplication analysis on *Populus trichocarpa* and *E. guineensis* using gene sequences and SSR markers, respectively (Fig. 4). As a result, 17,999 duplication events were identified in *Populus trichocarpa* using gene sequences. Many more duplication events (368,946) were obtained through SSRs. An overlap between gene-based and SSR-based duplication events was found in chromosomes 1, 3, 5, 7, 8, and 10 (Fig. 4a,b). In total, 6.6% (24,483) of the SSR-based duplication events overlapped with 11.2% (2,006) of the gene-based events. In *E. guineensis*, 601 gene-based and 1,726,902 SSR-based duplication events were identified, and an overlap was found between 0.24% (4,092) of the SSR-based and 45.9% (276) of the gene-based events.

## Discussion

The development of molecular markers in tree species has long been limited because of the lack of genome sequences. Recently, substantial progress has been made in genome sequencing^16–20^. Based on currently available data, we performed the genome-wide development of SSR, ILP, and PIP markers in 16 tree species, identifying a total of 524,170 SSR, 267,636 ILP, and 11,872 PIP markers. We found that the genome size was positively correlated with the number of SSR loci, but negatively correlated with their density. Consistently, the number of SSR markers showed a positive correlation with the genome size.

A recent study revealed the novel distribution pattern of SSRs in grass genomes^21^. Interestingly, short motifs including dimers, monomers, and trimers were the most abundant SSR types, which is the opposite of our observation in tree species. This may reflect evolutionary divergences between tree and grass species. However, common features were also observed between SSRs of trees and grasses. For instance, most SSRs were located in the intergenic regions of both tree and grass species. Moreover, although grass genomes are G/C rich, the sequences in grass SSR motifs did not show a similar pattern. This correlates with the finding that A/T bases comprised a much larger proportion than G/C bases in the SSR loci of tree species.

We analysed the distribution pattern of SSR and ILP markers on four scaffolds of the *M. notabilis* genome (Fig. 1a). This showed that the markers were alternatively distributed, suggesting their combined use would be highly efficient. This was further confirmed by PCR analysis (Fig. 3c). Most SSRs were monomorphic markers (Supplementary Table S6). In accordance with front studies, the proportions of polymorphic markers were positively correlated with the genome size (Supplementary Table S7), which can be explained by the increased number of binding sites in larger genomes.

To examine the efficiency of SSR and ILP markers identified in the present study, we performed a phylogenetic analysis of 30 willow samples and duplication analysis in *Populus trichocarpa* and *E. guineensis*. Because our results revealed an alternative distribute pattern between SSR and ILP markers, the phylogenetic analysis was performed using a combination of five SSR and six ILP markers. The 30 willow samples derived from seven provinces across China (Fig. 3a). Three samples located relatively close together in southwest China were clustered together, while 19 of 21 samples from Shandong province were clustered in the same group (Fig. 3b). These results suggest a positive correlation between genetic diversity and geographic distance. However, in Group V and Group VI, two samples far apart from each other were clustered together. We hypothesize that this may be because willows are prone to interspecific hybridization and interregional transition^22^.

Genome duplication is responsible for shaping the architecture and function as well as the evolution of many higher plant genomes, and gives rise to new or modified gene functions^23–25^. Therefore, analysing genome duplication is important for understanding the mechanism underlying evolution and gene functions. Duplication analysis had previously been studied in *Populus trichocarpa* and *E. guineensis* ^26,27^, although these were mainly based on gene coding sequence data. In the present study, we determined whether SSRs could be used for duplication analysis by performing this on *Populus trichocarpa* and *E. guineensis*. Together with previous findings, we found that most of *E. guineensis* were represented by segmental duplications, not triplications. We also identified a much larger number of duplications events using SSRs than gene coding sequences, and revealed a limited overlap between gene-based and SSR-based duplication events. Abundant microduplications were found based on SSR markers which mainly reflected the duplication events in the intergenic regions. These results suggest that SSRs are suitable for use in duplication analysis.

## Materials and Methods

### Data sources

The 16 tree species involved in this study were: *A. trichopo, E. guineensis, H. brasiliensis, J. curcas L., M. notabilis, Phoenix dactylifera, Pinus taeda L., Populus euphratica Oliv, Populus trichocarpa, Prunus persica, T. cacao L., S. babylonica, Pinus lambertiana, Picea abies, G. biloba L., and Fraxinus excelsior*. Genomes of 16 species were downloaded from public databases (Supplementary Table S8). Genomes from the model plants *Arabidopsis* and *Oryza sativa* were downloaded from the *Arabidopsis* Information Resource (https://www.arabidopsis.org/) and the Rice Genome Annotation Project (http://rice.plantbiology.msu.edu/), respectively.

### Development of SSR, ILP, and PIP markers

**Figure 5:**
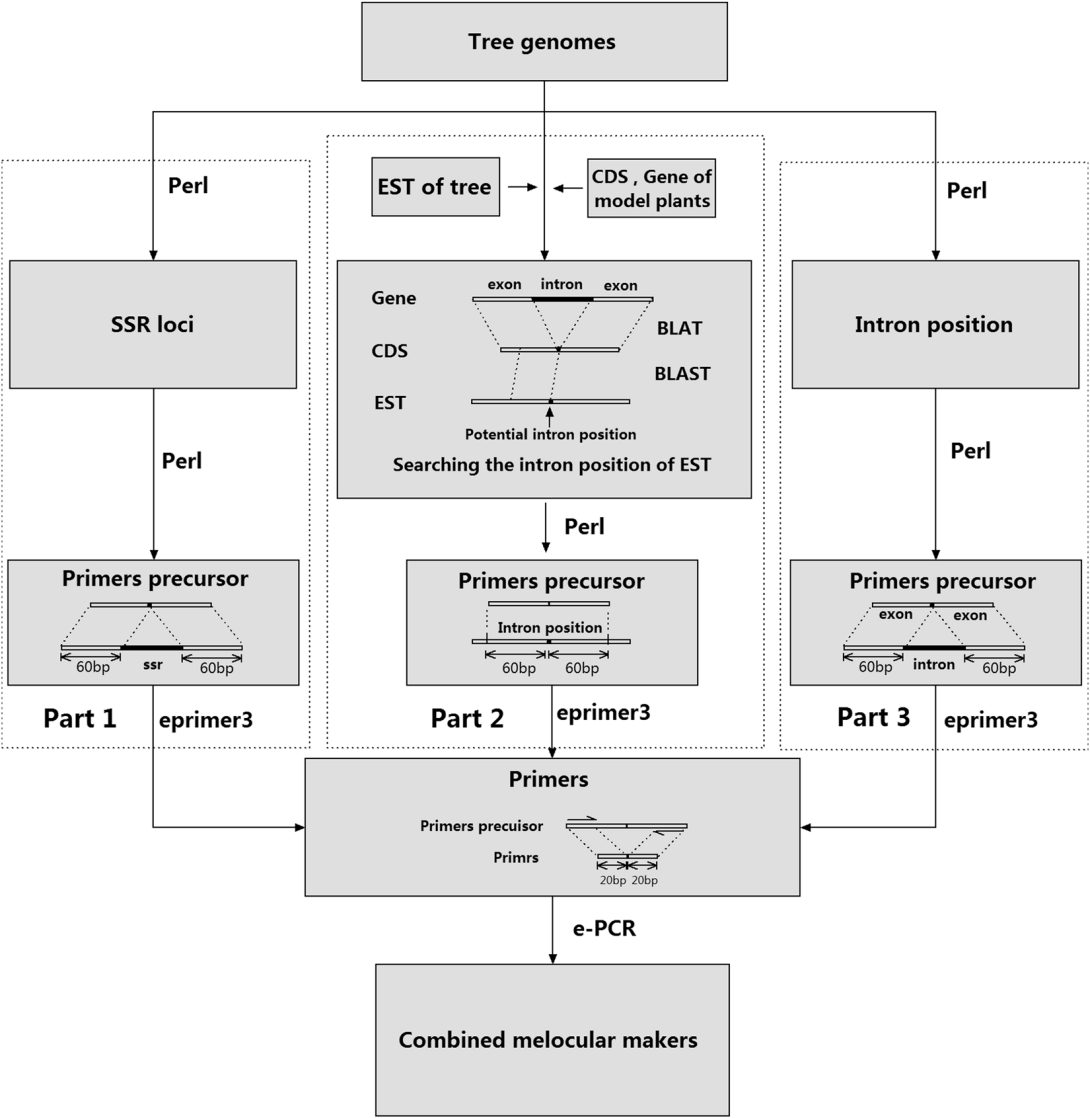
Flowchart of the development of SSR, PIP, and ILP markers. Part 1: SSR pipeline; Part 2: PIP pipeline; Part 3: ILP pipeline.

A pipeline composed of Perl scripts was used to search for SSR loci, based on 16 tree genomes. SSRs were classified into seven types: monomers (≥12 repeats), dimers (≥six repeats), trimers (≥four repeats), tetramers (≥three repeats), pentamers (≥three repeats), hexamers (≥two repeats), and heptamers (≥two repeats). Considering the principles of Watson–Crick base pairing and the initial motif position, some motifs were identified as one type of SSR locus. For instance, we identified AC, CA, TG, and GT as the SSR motif AC. A pair of 60-bp primer precursors flanking the SSR locus was cut to prepare for primer designing (Fig. 5, Part 1). For *Pinus taeda* and *Pinus lambertiana* in which only ESTs were available, the intron position information was unknown. Therefore, we developed PIP markers for these species by comparing available EST sequences with the genome sequences of the model plants *Arabidopsis* and *O. sativa*. As shown in Fig. 5, Part 2, the first step of this process was to find the intron positions of the model species by aligning its coding sequences (CDS) with its genome sequence using BLAT^28^. The second step was to identify potential intron positions by aligning EST sequences with the CDS of model species using BLAST^29^. The third step was to develop primers that flanked potential intron positions.

Perl scripts were used to extract exact intron positions for the tree species with complete genome data, and to select a pair of 60-bp primer precursors flanking each intron to identify ILP markers (Fig. 5, Part 3). Coupled primer pairs were designed by Windows-based Emboss: eprimer3^30^, based on the primer precursors we identified flanking the introns (ILP and PIP) and SSRs. The primers were tested using electronic PCR^31^ (e-PCR) against the corresponding genomes. A pair of primers was identified as a good molecular marker if it successfully amplified the desired fragment by e-PCR. Two markers were identified as the same if the forward or reverse primer was identical. A special Perl script was written to remove duplicated markers. All Perl scripts used in this study are available at http://biodb.sdau.edu.cn/xxyssr/result_data.zip.

### Distribution of SSR and ILP markers

Four DNA scaffolds containing the *M. notabilis* SSR and ILP markers were randomly selected to draw a distribution diagram using the R Language. DNA scaffolds with GenBank accession numbers NW_010356728.1, NW_010356865.1, NW_010358179.1, and NW_010359376.1 were renamed Scaffold 1–4, respectively. Each short vertical bar on the map represents the position of an SSR or ILP marker. The number of molecular markers (SSR or ILP) was counted using a Perl script and the molecular density (per Mb) of each scaffold was calculated. Based on the position, the number of concomitant and separated markers (SSR and ILP markers) was calculated for each tree species.

### Experimental verification of universal markers and diversity analysis of Chinese willows

All obtained markers were selected and checked against the genomes of 16 species via e-PCR. A Perl script was used to select universal markers that could amplify the fragments in all 16 species. To assess the marker performance, two primer pairs from each of universal SSR markers, universal ILP markers, and universal PIP markers (Supplementary Table S9) were randomly selected, then amplified in four species: *S. babylonica*, *Populus trichocarpa*, *M. notabilis*, and *Selaginella*. Furthermore, five SSR primer pairs and six ILP primer pairs of willow (Supplementary Table S10) were amplified in 30 different willow materials (Supplementary Table S11). The 30 willow samples were all from *S. babylonica*. To mark the sampling sites, a skeleton map was constructed by R package “maps” (https://cran.r-project.org/web/packages/maps/), then modified using Adobe Photoshop (version 14.0, X64). All primers were synthesised by Shanghai Sangon Biological Engineering & Technology Company.

DNA from the 30 willow materials and young leaves of other species was extracted using the CTAB method^32^. PCR reactions were performed in a total volume of 15 µl containing 20 ng template DNA, 0.36 µM of each primer, 0.25 mM of each dNTP, 2.5 mM MgCl_2_, 1 U Taq DNA polymerase, and 2.0 µL of 10× PCR buffer. PCR conditions were as follows: 4 min at 94 °C, followed by 35 cycles of 1 min at 94 °C, 1 min at 55 °C, 1 min at 72 °C, and a final extension for 10 min at 72 °C. Electrophoresis on a 6% non-denaturing polyacrylamide gel was used to separate the PCR products, and DNA bands were visualised by silver staining. A binary matrix was constructed in which every band position was scored as either present (1) or absent (0), based on our electrophoretogram of combined markers (five pairs of SSR markers and six pairs of ILP markers) amplified in the 30 willow materials. An UPGMA-based phylogenetic tree of the 30 willow materials was then estimated using NTSYSpc^33^ version 2.1.

### Proportion of polymorphic markers and duplication analysis

We randomly selected 20,000 SSR markers of 16 species to be electronically amplified against their own genomes. The number of amplification sites was calculated by the Perl program. A monomorphic marker was confirmed if it could only amplify one site, and a polymorphic marker as one that could amplify two or more sites. The number of these two types of markers was shown schematically using R language.

*Populus trichocarpa* and *E. guineensis* were selected for duplication analysis because of their well-characterised genomes. The protein sequences and SSR markers of the two species were first prepared, and the protein sequences compared against themselves by BLAST analysis, and SSR markers selected for e-PCR against their own genomes. Based on protein BLAST results and corresponding gff files, gene-based duplications were obtained using MCScanX^34^. According to the collinearity format results, duplicate blocks within the whole genome were linked by curved ribbons using Circos^35^. To obtain marker-based duplications, e-PCR results were modified into the BLAST format.

## Electronic supplementary material

Supplementary tables

## References

[1] Bonan GB (2008). Forests and climate change: forcings, feedbacks, and the climate benefits of forests. Science.

[2] Neale DB, Kremer A (2011). Forest tree genomics: growing resources and applications. Nature Reviews Genetics.

[3] Lau, N. S. *et al*. The rubber tree genome shows expansion of gene family associated with rubber biosynthesis. Scientific Reports 6 (2016). 10.1038/srep28594 PMC5008842 27339202

[4] Silva D. V. *et al*. Comparative evaluation of total RNA extraction methods in Theobroma cacao using shoot apical meristems.Genetics and molecular research: GMR, **15** (1) (2016). 10.4238/gmr.15017364 26985935

[5] Eibach R, Zyprian E, Welter L, Töpfer R (2015). The use of molecular markers for pyramiding resistance genes in grapevine breeding.VITIS-. Journal of Grapevine Research.

[6] Labbé J (2011). Survey and analysis of simple sequence repeats in the Laccaria bicolor genome, with development of microsatellite markers. Current genetics.

[7] Targońska M, Bolibok-Brągoszewska H, Rakoczy-Trojanowska M (2016). Assessment of genetic diversity in Secale cereale based on SSR markers. Plant Molecular Biology Reporter.

[8] Badoni, S. *et al*. Genome-wide generation and use of informative intron-spanning and intron-length polymorphism markers for high-throughput genetic analysis in rice. *Scientific reports* **6** (2016). 10.1038/srep23765 PMC4817136 27032371

[9] Wei H, Fu Y, Arora R (2005). Intron-flanking EST–PCR markers: from genetic marker development to gene structure analysis in Rhododendron. Theoretical and applied genetics.

[10] Zhao XQ, Wu WR (2008). Construction of a genetic map based on ILP markers in rice. (Zhongguo yi chuan xue hui bian ji).

[11] Kita T (2016). Development of intron length polymorphism markers in genes encoding diketide-CoA synthase and curcumin synthase for discriminating Curcuma species. Food chemistry.

[12] Yang L (2007). PIP: a database of potential intron polymorphism markers. Bioinformatics.

[13] Verde I (2013). The high-quality draft genome of peach (Prunus persica) identifies unique patterns of genetic diversity, domestication and genome evolution. Nature genetics.

[14] Albert VA (2013). The Amborella genome and the evolution of flowering plants. Science.

[15] Tang, C. *et al*. The rubber tree genome reveals new insights into rubber production and species adaptation. *Nature plants* (2016). 10.1038/nplants.2016.73 27255837

[16] Motamayor, J C. *et al*. The genome sequence of the most widely cultivated cacao type and its use to identify candidate genes regulating pod color. *Genome biology* 14.6 r53 (2013). 10.1186/gb-2013-14-6-r53 PMC4053823 23731509

[17] Dai X (2014). The willow genome and divergent evolution from poplar after the common genome duplication. Cell research.

[18] Sato S (2010). Sequence analysis of the genome of an oil-bearing tree, Jatropha curcas L. DNA research.

[19] Nystedt B (2013). The Norway spruce genome sequence and conifer genome evolution. Nature.

[20] Sollars ESA (2017). Genome sequence and genetic diversity of European ash trees. Nature.

[21] Wang, X. & Wang, L. GMATA: an integrated software package for genome-scale SSR mining, marker development and viewing. *Frontiers in plant science* 7 (2016). 10.3389/fpls.2016.01350 PMC5020087 27679641

[22] Karp A (2011). Genetic improvement of willow for bioenergy and biofuels free access. Journal of integrative plant biology.

[23] Berthelot, C. *et al*. The rainbow trout genome provides novel insights into evolution after whole-genome duplication in vertebrates. *Nature communications* **5** (2014). 10.1038/ncomms4657 PMC4071752 24755649

[24] Glasauer SMK, Stephan CFN (2014). Whole-genome duplication in teleost fishes and its evolutionary consequences. Molecular genetics and genomics.

[25] Suzuki H (2015). Distinct functions of two olfactory marker protein genes derived from teleost-specific whole genome duplication. BMC evolutionary biology.

[26] Ma T (2013). Genomic insights into salt adaptation in a desert poplar. Nature communications.

[27] Singh R (2013). Oil palm genome sequence reveals divergence of interfertile species in Old and New worlds. Nature.

[28] Kent WJ (2002). BLAT—the BLAST-like alignment tool. Genome research.

[29] Altschul SF, Gish W, Miller W, Myers EW, Lipman DJ (1990). Basic local alignment search tool. Journal of molecular biology.

[30] Rice, P., Longden, I. & Bleasby, A. EMBOSS: the European molecular biology open software suite (2000). 10.1016/s0168-9525(00)02024-2 10827456

[31] Schuler GD (1998). Electronic PCR: bridging the gap between genome mapping and genome sequencing. Trends in biotechnology.

[32] Murray MG, Thompson WF (1980). Rapid isolation of high molecular weight plant DNA. Nucleic acids research.

[33] Rohlf, F. J. NTSYS-pc: numerical taxonomy and multivariate analysis system. *Applied Biostatistics* (1992).

[34] Wang Y (2012). MCScanX: a toolkit for detection and evolutionary analysis of gene synteny and collinearity. Nucleic acids research.

[35] Krzywinski (2009). Circos: an information aesthetic for comparative genomics. Genome research.

