# Supplementary material for: Genome-wide analysis of SSR and ILP markers in trees: diversity profiling, alternate distribution, and applications in duplication
Source: Sci Rep. 2017 Dec 20;7:17902. doi: 10.1038/s41598-017-17203-6 (PMC5738346; doi:10.1038/s41598-017-17203-6)
Supplement: Supplementary file 1 — Supplementary tables [file 41598_2017_17203_MOESM1_ESM.pdf]

SUPPLEMENTARY INFORMATION

Genome-wide analysis of SSR and ILP markers in trees: diversity profiling, alternate distribution, and applications in duplication

Xinyao Xia[1,3], Lin Lin Luan[1,3], Guanghua Qin[2,3], Li Fang Yu[1], Zhi Wei Wang[1], Wan Chen Dong[1], Yumin Song[2], Yuling Qiao[2], Xian Sheng Zhang[1], Ya Lin Sang[1]* and Long Yang[2]*

1 College of Forestry, College of Plant Protection, College of Life Sciences, State Key Laboratory of Crop Biology, Agricultural Big-Data Research Center, Shandong Agricultural University, Tai'an 271018, China
2 Shandong Academy of Forestry, Jinan, 250014, China
3 These authors contributed equally to this work
* Corresponding authors: Long Yang and Ya Lin Sang
Tel: +86 538 8246020; Fax: +86538-8226399. E-mail: lyang@sdau.edu.cn, sangyl@sdau.edu.cn

Supplementary Table S1. Details of seven types motifs in 16 tree species

Motif<br>Species	Monomer	Dimer	Trimer	Tetramer	Pentamer	Hexamer	Heptamer	All	Size	Density
Prunus persica	27,034	40,294	23,975	31,782	12,441	178,105	58,671	372,302	219	1700
	7.26%	10.82%	6.44%	8.54%	3.34%	47.84%	15.76%	100.00%		
Salix babylonica	31,055	57,451	51,276	50,049	20,208	254,705	86,345	551,089	295	1868
	5.64%	10.42%	9.30%	9.08%	3.67%	46.22%	15.67%	100.00%		
Jatropha curcas	83,074	33,951	32,392	42,609	12,737	227,431	78,032	510,226	308	1657
	16.28%	6.65%	6.35%	8.35%	2.50%	44.57%	15.29%	100.00%		
Morus notabilis	111,688	72,963	42,208	48,430	24,611	279,875	128,953	708,728	312	2272
	15.76%	10.29%	5.96%	6.83%	3.47%	39.49%	18.19%	100.00%		
Theobroma cacao	32,673	35,792	30,515	38,622	14,538	250,827	80,076	483,043	334	1446
	6.76%	7.41%	6.32%	8.00%	3.01%	51.93%	16.58%	100.00%		
Populus trichocarpa	59,920	54,354	57,563	60,302	26,344	336,295	124,341	719,119	403	1784
	8.33%	7.56%	8.00%	8.39%	3.66%	46.76%	17.29%	100.00%		
Populus euphratica	63,381	65,147	118,408	82,122	29,200	432,937	153,442	944,637	480	1968
	6.71%	6.90%	12.53%	8.69%	3.09%	45.83%	16.24%	100.00%		
Phoenix dactylifera	35,953	61,436	49,823	55,589	21,096	324,903	103,674	652,474	547	1193
	5.51%	9.42%	7.64%	8.52%	3.23%	49.80%	15.89%	100.00%		
Amborella trichopo	100,331	159,465	65,405	69,895	26,679	469,772	185,400	1,076,947	682	1579
	9.32%	14.81%	6.07%	6.49%	2.48%	43.62%	17.22%	100.00%		
Fraxinus excelsior	109979	52036	54512	84842	37870	499268	169920	1008427	846	1192
	10.91%	5.16%	5.41%	8.41%	3.76%	49.51%	16.85%	100.00%		
Hevea brasiliensis	78,165	103,931	107,094	154,575	69,316	827,752	280,723	1,621,556	1,362	1191
	4.82%	6.41%	6.60%	9.53%	4.27%	51.05%	17.31%	100.00%		
Elaeis guineensis	78323	105,403	85,834	108,984	43,820	747,818	265,081	1,435,263	1,485	967
	5.46%	7.34%	5.98%	7.59%	3.05%	52.10%	18.47%	100.00%		
Ginkgo biloba	356257	893397	319060	747079	99851	3423774	1065480	6904898	10220	676
	5.16%	12.94%	4.62%	10.82%	1.45%	49.58%	15.43%	100.00%		
Picea abies	333417	227946	306955	341052	100714	2501615	805669	4617368	11980	385
	7.22%	4.94%	6.65%	7.39%	2.18%	54.18%	17.45%	100.00%		
Pinus taeda	570,852	840,323	1,399,969	1,507,006	401,640	10,793,943	3,524,504	19,038,237	21709	877
	3.00%	4.41%	7.35%	7.92%	2.11%	56.70%	18.51%	100.00%		
Pinus lambertiana	862648	1435961	1701344	1895896	625256	15553791	4618933	26693829	27238	980
	3.23%	5.38%	6.37%	7.10%	2.34%	58.27%	17.30%	100.00%		
Total	2,934,751	4,239,851	4,446,334	5,318,835	1,566,321	37,102,818	11,729,247	67,338,158	78,420	
Percentage	4.36%	6.30%	6.60%	7.90%	2.33%	55.10%	17.42%	100%		

Supplementary Table S2. Statistics of the number of single base among the scanned SSR loci in 16 tree species.

**Species**	**A**	**T**	**G**	**C**	**AT/GC**
P. persica	654179	654905	255532	256557	2.56
S. babylonica	1584771	1080477	279507	797924	2.47
J. curcas	913838	917598	238863	239710	3.83
M. notabilis	1232878	1218322	349232	370814	3.40
T. cacao	965458	963933	262381	264029	3.67
P. trichocarpa	1406641	1411834	361418	362589	3.89
P. euphratica	1909181	1902548	410688	409305	4.65
P. dactylifera	1109076	1113022	524370	528845	2.11
A. trichopo	1818918	1839284	690203	696147	2.64
F. excelsior	2141946	2255624	1171874	951598	2.07
H. brasiliensis	3329764	3337973	881612	876248	3.79
E. guineensis	2758663	2760411	973106	973639	2.84
G. biloba	19975995	13084675	4202813	11098101	2.16
P. abies	8683774	8684160	3422690	3436716	2.53
P. taeda	36733099	35944354	15244918	15429592	2.37
P. lambertiana	52627165	52349397	19624393	19740140	2.67

Supplementary Table S3. Statistics of the ILP loci.

Specis	genome size(Mb)	ILP loci	Density(number/Mb)
P. persica	219	232758	1063
S. babylonica	295	235984	800
J. curcas	308	346151	1124
M. notabilis	312	193575	620
T. cacao	334	505195	1513
P. trichocarpa	403	375440	932
P. euphratica	480	656824	1368
P. dactylifera	547	498814	912
A. trichopo	682	240240	352
F. excelsior	846	null	nul
H. brasiliensis	1362	null	null
E. guineensis	1485	526379	354
G. biloba	10220	null	null
P. abies	11981	null	null
P. taeda	21709	null	null
P. lambertiana	26931	null	null

Supplementary Table S4. Statistics of the SSR, ILP and PIP markers. PIP-Ath and PIP-Rice represent the number of potential intron polymorphism markers which were obtained by comparing with the Arabidopsis and rice.

Species	SSR Markers	ILP Markers	PIP-Ath	PIP-Rice
P. persica	27,139	11,832		
S. suchowensis	22,820	13,460		
J. curcas	21,442	21,089		
M. notabilis	29,082	12,266		
T. cacao	22,892	29,672		
P. trichocarpa	33,371	25,289		
P. euphratica	37,028	43,374		
P. dactylifera	52,001	33,980		
A. trichopo	43,040	15,813		
F. excelsior	26,444	null		
H. brasiliensis	64,913	null	1,260	453
E. guineensis	70,442	35,545		
G. biloba.	20,000	25,316		
P. abies	20,000	null		
P. taeda	20,000	null	9,441	718
P. lambertiana	20,000	null		
In total	530,614	267,636	10,701	1,171

Supplementary Table S5. Statistics of the intersectant and separated markers in 10 tree species.

**Species**	**Number of SSR markers**	**Numbers of ILP markers**	**Number of intersectant markers**	**Ratio**
P. persica	27,139	11,832	637	2.35%
S. babylonica	22,820	13,460	826	3.62%
J. curcas	21,442	21,089	1117	5.21%
M. notabilis	29,082	12,266	758	2.61%
T. cacao	22,892	29,672	1593	6.96%
P. trichocarpa	33,371	25,289	1300	3.90%
P. euphratica	37,028	43,374	2548	6.88%
P. dactylifera	52,001	33,980	4214	8.10%
A. trichopo	43,040	15,813	1730	4.02%
E. guineensis	70,442	35,545	4344	6.17%

Supplementary Table S6. Proportion of the monomorphic and polymorphic markers in 16 tree species. If a marker only amplified one site and it would be defined as monomorphic marker. Polymorphic markers could amplified two or more sites. .

**The number of amplyfied sites**<br>**Species**	**1**	**2**	**3**	**4**	**5**	**others**
P. persica	84.3200%	6.0950%	2.4350%	1.1250%	0.6800%	5.3450%
S. babylonica	89.1980%	6.6810%	1.7100%	0.7750%	0.4100%	1.2260%
J. curcas	81.5240%	6.2390%	2.6370%	1.5700%	1.1750%	6.8550%
M. notabilis	79.5850%	7.9690%	2.7480%	1.4640%	1.0230%	7.2110%
T. cacao	78.9790%	4.6950%	1.9350%	1.4100%	0.9850%	11.9960%
P. trichocarpa	84.9690%	7.3050%	1.9150%	0.9200%	0.5250%	4.3660%
P. euphratica	79.7600%	9.0050%	1.4750%	0.6500%	0.4150%	8.6950%
P. dactylifera	83.3480%	7.2270%	1.9580%	1.0870%	0.5260%	5.8540%
A. trichopo	78.6530%	8.2410%	2.9260%	1.6980%	1.2370%	7.2450%
F. excelsior	91.8880%	6.0960%	1.0000%	0.4000%	0.2250%	0.3910%
H. brasiliensis	59.0150%	8.3300%	3.4120%	2.2900%	1.5270%	25.4260%
E. guineensis	81.1720%	6.7360%	2.3050%	1.2400%	0.8400%	7.7070%
P. abies	75.2760%	6.4570%	2.9060%	1.7210%	1.2500%	12.3900%
Norway spruce	77.4120%	10.9890%	2.9110%	1.4360%	0.9900%	6.2620%
P. taeda	47.3000%	8.0200%	4.0930%	2.5400%	2.1540%	35.8930%
P. lambertiana	36.5020%	7.7700%	4.2150%	2.8700%	2.1250%	46.5180%

Supplementary Table S7. Statistics of the proportion of polymorphic markers and genome repeatability rates in 16 tree species.

**Specis**	**genome size(Mb)**	**Proportion of polymorphic markers**	**Genome repeatability rate**
P. persica	219	15.68%	30%
S. babylonica	295	10.80%	null
J. curcas	308	18.48%	37%
M. notabilis	312	20.42%	47%
T. cacao	334	21.02%	42%
P. trichocarpa	403	15.03%	47%
P. euphratica	480	20.24%	44%
P. dactylifera	547	16.65%	null
A. trichopo	682	21.35%	57%
F. excelsior	846	8.11%	36%
H. brasiliensis	1362	40.99%	71%
E. guineensis	1485	18.83%	57%
G. biloba	10220	24.72%	77%
P. abies	11981	22.59%	70%
P. taeda	21709	52.70%	82%
P. lambertiana	26931	63.50%	86%

Supplementary Table S8. Download links of the 16 tree genomes.

Tree species	Download website	Download Date
A. trichopo	http://www.ncbi.nlm.nih.gov/genome/?term=Amborella+trichopo	2016/5/25
E. guineensis	http://www.ncbi.nlm.nih.gov/genome/?term=Elaeis+guineensis	2016/5/25
J. curcas	http://www.ncbi.nlm.nih.gov/genome/?term=Jatropha%20curcas%5Borgn%5D	2016/5/25
M. notabilis	http://www.ncbi.nlm.nih.gov/genome/?term=Morus+notabilis	2016/5/25
P. dactylifera	http://www.ncbi.nlm.nih.gov/genome/?term=Phoenix%20dactylifera	2016/5/25
P. euphratica	http://www.ncbi.nlm.nih.gov/genome/13265	2016/5/25
P. trichocarpa	http://www.ncbi.nlm.nih.gov/genome/98	2016/5/25
P. persica	http://www.ncbi.nlm.nih.gov/genome/?term=Prunus%20persica	2016/5/25
T. cacao L.	http://www.ncbi.nlm.nih.gov/genome/?term=Theobroma%20cacao	2016/5/25
H. brasiliensis	http://www.ncbi.nlm.nih.gov/genome/?term=Hevea%20brasiliensis	2016/5/25
P. taeda	http://www.ncbi.nlm.nih.gov/genome/?term=Pinus+taeda	2016/5/25
S. babylonica	http://115.29.234.170/willow/	2016/5/25
P. lambertiana	http://www.ncbi.nlm.nih.gov/genome/12196	2016/5/25
P. abies	http://congenie.org/downloads	2017/3/5
G. biloba L.	http://gigadb.org/dataset/100209	2017/3/5
F. excelsior	https://www.ncbi.nlm.nih.gov/genome/?term=European+ash	2017/3/5

Supplementary Table S9. Details of the 6 universal SSR,ILP,and PIP makers.

**Makers**	**Forward primers**	**Reverse primers**
USSR1	TCGACGATGATGACGATT	TCATCATCACCAACAGCAG
USSR2	CATCAGCAGCAACAGCAG	TGATCGGCACCAGCTCTA
UILP1	GTCACCTGGAACCAGCAA	TTCATGCATCCTCTTCCTT
UILP2	TGCCATGGAAGAAGAAGAAGA	TCGATCGCATTCCTTCCT
UPIP1	CTCGGACACCATCGACAA	GGCCATCTTCGAGCTGTT
UPIP2	CTGACTGGCAAGACCATCAC	TGCTGATCCGGAGGAATC

Supplementary Table S10. Details of the 5 SSR makers and 6 ILP makers from willow for experiments.

**Makers**	**Forward primers**	**Reverse primers**
SSR1	AGAGCTTCAACGGAGGATACC	AACAGTACCGCCACCTTCC
SSR2	AATCAACACACATCCTCCATGT	GCAAGTTGATGAGCTGATGG
SSR3	AGAAGCCACCATGGCTAGA	AAGTTGTCTCTCCACCTTCG
SSR4	GCCATGTATTGCATAGACAGAGA	TAGGTTCGATTGGCGGATA
SSR5	ATATGCGATCCAACCATCAA	TCTTGAAGTCCACCTTAGCC
ILP1	TCTTCTTGGTTCTGCTCTGCT	CCTCATCTGTTGTTCCTATTATCTC
ILP2	GAAGCAGATGGTGCTTCCTAA	CCAATTCAAGCATGGCATAA
ILP3	GAGAGAGCACAGGCTCGTT	TTGTCTTCTCAAGTTCTTCACC
ILP4	TTACATCAGACTTGCTGCTTCTG	AATCAGAATTGCTGGAATAGCC
ILP5	TTGATGCCTGACTCGTCTAA	ACCACTCCGAAGCTATAGACA
ILP6	CCAGCAACATTCTGTTAGATGA	CTTGACCGAGAAGTGTCCTTG

Supplementary Table S11. Details of the 30 willow materials.

Samples Name	Longitude and Latitude of Sampling Position	General Location
GHL	N34° 16'56.24" E108° 4'27.95"	Shaanxi province
CQL	N29° 48'38.48" E106° 23'26.18"	Chongqing
SY	N32° 24.226′ E110° 42.246′	Hubei province
GSHL	N36° 03'48.32" E103° 50'26.50"	Gansu province
YL	N32° 03'40.73" E118° 47'10.23"	Jiangsu province
HBL	N30° 42'29.37" E111° 17'52.78"	Hubei province
Sh2	N33° 59.842′ E108° 49.806′	Shaanxi province
Sh1	N33° 59.842′ E108° 49.806′	Shaanxi province
HZL	N30° 15'25.25" E120° 08'1.43"	Zhejiang province
HK1	N37° 57.480´ E118° 19.818´	Shandong province
HK2	N37° 57.212´ E118° 20.288´	Shandong province
ZH1	N37° 40.400´ E118° 05.800´	Shandong province
HM1	N37° 14.381´ E117° 31.359´	Shandong province
SX1	N34° 36.607´ E116° 00.215´	Shandong province
MY2	N35° 36.705´ E117° 59.873´	Shandong province
DY1	N37° 45.299´ E119° 06.929´	Shandong province
ZB2	N36° 47.950´ E117° 55.092´	Shandong province
BZ4	N37° 27.199´ E118° 05.910´	Shandong province
SH13	N37° 20.94´ E117° 10.73´	Shandong province
HM3	N37° 19.750´ E117° 27.919´	Shandong province
KL1	N37° 32.950´ E118° 19.496´	Shandong province
WL1	N37° 45.200´ E117° 37.460´	Shandong province
DY2	N37° 45.300´ E119° 06.930´	Shandong province
BZ3	N37° 27.198´ E118° 05.909´	Shandong province
YZ1	N35° 33'46.67" E116° 43'45.20"	Shandong province
SS2	N35° 40'34.98" E117° 13'43.02"	Shandong province
SS1	N35° 40'34.98" E117° 13'43.02"	Shandong province
ZH2	N37° 39.138´ E118° 04.827´	Shandong province
B88	N37° 21'4.35" E117° 57'58.84"	Shandong province
ZB1	N36° 47.907´ E117° 54.697´	Shandong province

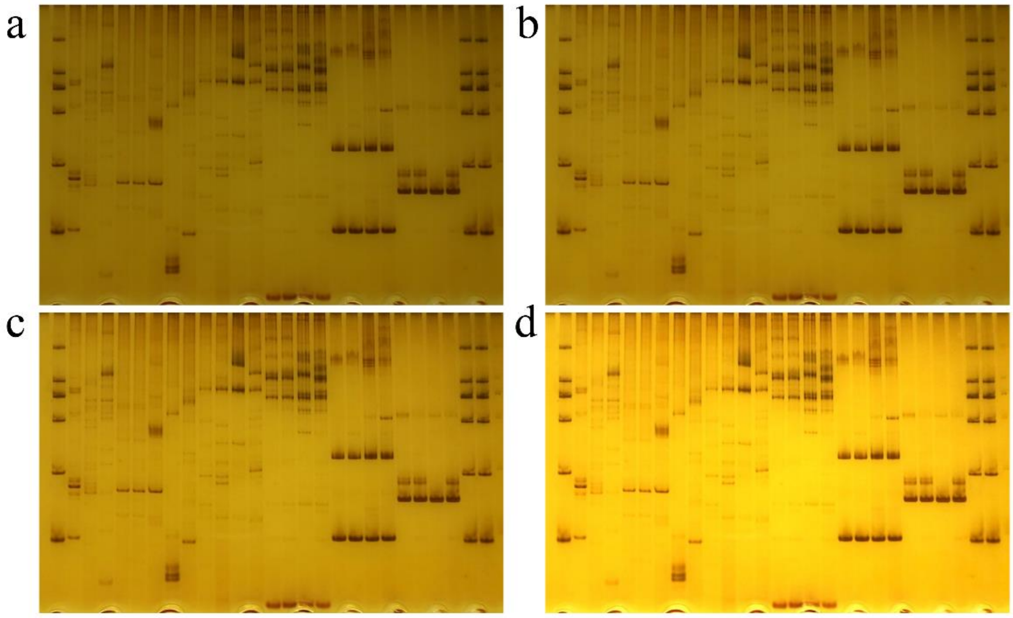

Supplementary Information. Full-length gels with different exposures. (a)Original image with no modification of exposure. (b)Exposure value +0.5. (c) Exposure value +1. (d) Exposure value +2. All the exposures of these pictures were modified by Adobe Photoshop (version 14.0, x64).
